# Volatile Components and Preliminary Antibacterial Activity of Tamarillo (*Solanum betaceum* Cav.)

**DOI:** 10.3390/foods10092212

**Published:** 2021-09-17

**Authors:** Tung Thanh Diep, Michelle Ji Yeon Yoo, Chris Pook, Saeedeh Sadooghy-Saraby, Abhishek Gite, Elaine Rush

**Affiliations:** 1 School of Science, Faculty of Health and Environment Sciences, Auckland University of Technology, Private Bag 92006, Auckland 1142, New Zealand; tung.diep@aut.ac.nz (T.T.D.); saeedeh.sadooghy-saraby@aut.ac.nz (S.S.-S.); giteabhi27@gmail.com (A.G.); 2 Centre of Research Excellence, Riddet Institute, Massey University, Private Bag 11222, Palmerston North 4442, New Zealand; elaine.rush@aut.ac.nz; 3 The Liggins Institute, The University of Auckland, Private Bag 92019, Auckland 1142, New Zealand; chris.pook@auckland.ac.nz; 4 School of Sport and Recreation, Faculty of Health and Environment Sciences, Auckland University of Technology, Private Bag 92006, Auckland 1142, New Zealand

**Keywords:** freeze-dried tamarillo, TD–GC–MS, volatiles, antimicrobial activity

## Abstract

Tamarillo is a nutrient-dense fruit with a unique aroma from its volatile compounds (VCs). In this study, we aimed to compare the volatile profiles: (i) of fresh and freeze-dried tamarillo; (ii) detected using Thermal Desorption–Gas Chromatography–Mass Spectrometry (TD–GC–MS) and Solid-Phase MicroExtraction–Gas Chromatography-Mass Spectrometry (SPME–GC–MS); (iii) of freeze-dried pulp and peel of New Zealand grown tamarillo. The possible antibacterial activity of freeze-dried tamarillo extracts was also investigated. We show that freeze-drying maintained most of the VCs, with some being more concentrated with the loss of water. The most abundant VC in both fresh and freeze-dried tamarillo was hexanoic acid methyl ester for pulp (30% and 37%, respectively), and (E)-3-Hexen-1-ol for peel (36% and 29%, respectively). With the use of TD–GC–MS, 82 VCs were detected for the first time, when compared to SPME–GC–MS. Methional was the main contributor to the overall aroma in both peel (15.4 ± 4.2 μg/g DW) and pulp (118 ± 8.1 μg/g DW). Compared to water as the control, tamarillo extracts prepared by water and methanol extraction showed significant antibacterial activity against *E. coli*, *P. aeruginosa*, and *S. aureus* with zone of inhibition of at least 13.5 mm. These results suggest that freeze-dried tamarillo has a potential for use as a natural preservative to enhance aroma and shelf life of food products.

## 1. Introduction

Tamarillo (*Solanum betaceum* Cav.) is a good source of phenolics and anthocyanins with high antioxidant activity (52.42–60.19 μmol TEAC/g DW) [1]. It is cultivated in New Zealand, Ecuador, Brazil, and Colombia, and it is mostly consumed as fresh fruit [1]. Laird’s Large, also known as red type, is the most common cultivar. Tamarillo pulp contains high amounts of dietary fiber, vitamins A, B_6_, C, and E, and minerals (Ca, K, Cu, Fe, Mg, Mn, P, and Zn) [2,3,4,5]. Chlorogenic acid and kaempferol rutinoside are dominant phenolics in red tamarillo from New Zealand; high concentrations of delphinidin rutinoside and pelargonidin rutinoside were found in this variety [1]. Meanwhile, β-carotene and β-cryptoxanthin were identified as the most abundant carotenoids in tamarillo from Australia and Brazil [6] as well as New Zealand [3]. Potential health benefits including antioxidant, antiobesity, anticancer, and prebiotic properties of tamarillo fruit have been reported [5]. Recognized for a distinctive aroma, tamarillo pulp is mostly consumed in the fresh form and the peel is discarded. Aroma is one of the important parameters for the quality of fruit and fruit-derived products, with volatile compounds (VCs) determining the aroma component of flavor. Determination of volatile composition could explain various impacts of VCs on the aroma and aroma of food as well as the degree of acceptance of fruit by consumers. Dependent on the country of origin and cultivar, variations in volatile composition of mainly the mesocarp and endocarp components of fresh tamarillo pulp have been reported [6,7,8]. Freeze-drying is the most common nonthermal preservation technique for heat-sensitive fruit and vegetables [9] that enables maintenance of flavor volatiles [9], bioactivity, color, and nutrients [10] compared to conventional thermal processing methods. To the best of our knowledge, volatile profiles of freeze-dried tamarillo and the volatile profile of tamarillo peel (exocarp) remain unknown. Often, fruit peels (including tamarillo) are discarded as waste products; this may explain the lack of data.

Fruit extracts are known to exhibit antimicrobial activity [11]. Only one study has reported on positive effects of tamarillo extracts (invertase inhibitory protein) against the growth of xylophagous and phytopathogenic fungi and phytopathogenic bacteria [12]. They report that the invertase inhibitory protein concentration required to inhibit the growth of fungi and phytopathogenic bacteria is between 7.8 to 62.5 μg mL^−1^ and 7.8 to 31.25 μg mL^−1^, respectively. From Santose & de Aquino Santana [13], who reported on antimicrobial potential of exotic fruit residues, it remains unclear whether the antimicrobial activity of tamarillo is present in the pulp and/or in the peel and if the inhibitory action could be extended to other microorganisms of concern for foods. Therefore, the aims of the current study were to compare: (i) volatile profiles of fresh and freeze-dried pulp; (ii) volatile profiles of tamarillo detected by Thermal Desorption–Gas Chromatography–Mass Spectrometry (TD–GC–MS) or Solid-Phase MicroExtraction–Gas Chromatography-Mass Spectrometry (SPME–GC–MS); and (iii) volatile profiles of freeze-dried pulp and peel of tamarillo. We hypothesize that freeze-drying may cause some loss of VCs and that more VCs that were not previously detected before may be detected using TD–GC–MS, a highly sensitive technique. We also investigated the possible antibacterial activity of freeze-dried tamarillo extracts.

## 2. Materials and Methods

### 2.1. Sampling

Fresh fruit of Laird’s Large (red cultivar) tamarillo were collected from Northland region of New Zealand. Commercial maturity was between 21 and 24 weeks from anthesis. Thirty fruits were washed thoroughly with tap water, dried, separated into peel (exocarp) and pulp (mesocarp and endocarp), and then freeze-dried (Alpha 1–2 LD plus Freeze Dryer, Martin Christ, New Zealand) and ground to powder. To minimize the loss of volatiles, particularly those of low boiling points, the samples were deep frozen at −85 °C in the bowl of the freeze drier. The powder was stored in a freezer at −20 °C until further analysis. For the comparison experiment of volatile components between fresh and freeze-dried samples (Section 2.3), the peeled fresh fruit was quickly homogenized using a commercial stainless steel blender (SKU MBR-1101, Nutribullet, New Zealand) and then analyzed immediately.

### 2.2. Chemicals and Reagents

All chemicals and reagents used were AnalaR grade or better and obtained from Sigma-Aldrich (Sigma Aldrich Ltd., Auckland, New Zealand). Milli-Q water was produced using a Purite Fusion Milli-Q water purifying machine (Purite Limited, Thame, Oxon, UK).

### 2.3. Analysis of VCs of Tamarillo Fruits by SPME–GC–MS

Comparison between fresh and freeze-dried tamarillo was conducted to examine whether the loss of VCs was significant from the freeze-drying process. This experiment was carried out using solid-phase microextraction (SPME) and GC–MS. Samples (approximately 1 g of fresh pulp and 0.1 g of freeze-dried pulp) were quickly introduced into 10 mL headspace vial. A 2 μL of the internal standard was added into the vial. The head space vial was heated at 50 °C for 15 min using an incubator equipped with an agitator set at 250 rpm for better extraction. The SPME fiber was 50/30 μm Divinylbenzene/Carboxen/Polydimethylsiloxane (DVB/CAR/PDMS), StableFlex fiber 24 ga, length of 2 cm (Supelco, Bellefonte, PA, USA). The fiber was exposed to the sample headspace for 20 min and desorbed for 5 min at a desorption temperature of 50 °C.

Phenomenex ZB-1701 column was used to analyze the volatiles through GC–MS. Helium was the carrier gas with a constant flow rate of 1.1 mL min^−1^. The mode of injection was splitless, and the inlet temperature for the injection port was set to 250 °C with 45 mL min^−1^ split flow and 2 min splitless time. Chromatographic conditions were as follows: the oven was held at 40 °C for 2 min, then raised to 280 °C at a rate of 8 °C min^−1^ and held for 1.0 min. The total run time was 33 min. The MS was operated in the electron impact mode (EI) with a source temperature of 230 °C, a quadrupole temperature of 150 °C, an ionizing voltage of 70 eV, and a transfer line temperature of 250 °C. The MS scanned masses from 38 to 450 *m*/*z*.

### 2.4. Analysis of VCs of Tamarillo Fruits by TD–GC–MS

Between 1000 and 1200 µg of the freeze-dried, powdered sample of tamarillo was placed in a glass thermal desorption unit (TDU) insert on a Sartorius CPA2P microbalance (Weightec (NZ) Ltd., Auckland, New Zealand), massed to ±10 µg and then capped for GC–MS. Peel and pulp samples were separately analyzed. A gas chromatography system (Agilent 6890B GC and 5977B MSD, Agilent Technologies, Santa Clara, CA, USA) equipped with a Gerstel multipurpose sampler, thermal desorption (TD) unit, and cooled inlet system (CIS) were used to extract and analyze the volatiles. Helium was used as a carrier gas with a constant flow rate of 1.0 mL min^−1^. A Phenomenex ZB-1701 column (Phenomenex NZ, Auckland, New Zealand) measuring 30 m × 250 μm × 0.15 μm, with a 5 m guard, was used to separate polar and nonpolar VCs.

The autosampler was spiked with 2.0 µL of the internal standard (10.0 mg L^−1^ solution of 2-chlorophenol in water), added to each sample immediately prior to TD. The TD unit was run in solvent venting mode for 1.0 min at 40 °C, then switched to splitless extraction, and the temperature was increased at the rate of 65 °C min^−1^ to 150 °C and held for 4.0 min. In solvent-venting mode operation, the CIS trapped and cryofocused VCs at −40 °C with a helium flow of 80 mL min^−1^. Then, the GC–MS started, and the CIS was operated in splitless mode with a fixed helium flow rate of 1.1 mL min^−1^ and a septum purge flow of 3.0 mL min^−1^. Trapped VCs were released from the CIS by ballistic heating at 11 °C s^−1^ to 290 °C and held for 4.0 min. Then, the helium flow to the split vent was set to 45.0 mL min^−1^ for the rest of the run.

A preliminary test was carried out at different temperatures (90 °C, 120 °C, 150 °C, and 180 °C) to identify the optimal temperature for release of the volatiles. Based on the peak areas and separation of the peaks, 150 °C was found to be the optimal temperature. Chromatographic conditions were as follows: the oven was held at 40 °C for 2.0 min, then raised to 280 °C at a rate of 8 °C min^−1^ and held for 1.0 min, with a total run time of 33.0 min. The mass spectrometer was operated in electron impact mode (EI) with an electron voltage of 70 eV and transfer line temperature of 260 °C. The temperatures of 230 °C and 150 °C were set up for MS source and MS quadrupole, respectively. The mass spectrometer scanned from 43 to 450 *m*/*z* at a rate of 5.5 scans per second. Agilent MassHunter GC–MS software version B.09.00 (Agilent Technologies, Victoria, Australia) was used to process the data.

Another comparison test between fresh and freeze-dried tamarillo was conducted with fresh pulp (approximately 88% moisture content) [4] using TD–GC–MS at temperature of 50 °C in order to minimize the sugar artefact and oxidation. No-solvent delay mode was set up to ensure the detection of volatiles with low boiling points. All other parameters were kept the same as the main experiment described above. Approximately 10 mg of the fresh sample was kept in −20 °C and put in the GC–MS immediately prior to analysis to minimize enzymatic degradation.

Chromatographic peaks were deconvoluted and extracted using Agilent Unknowns software (Agilent Technologies, Victoria, Australia). Using each feature’s Kovats index as a secondary identifier, the 2014 National Institute of Standards and Technology (NIST) Mass Spectral Reference Library was searched for matches. To confirm the identity of each VC, linear retention indices (LRIs) were calculated for each VC using the retention times of a homologous series of *n*-alkanes series (C_7_–C_30_). The base peak *m*/*z* and retention time of the best match for each feature was then passed to MassHunter Quant B.07.00 software where peak areas of each feature in every sample were quantified relatively to that of the internal standard. Finally, the peak areas for each target were blank subtracted and normalized to the sample mass.

### 2.5. Determination of Odor Threshold and Relative Odor Activity Value (OAV)

Odor threshold values from tomato (the same *Solanum* genus) [14] and odor threshold values published by Leffingwell & Associates [15] were used to determine the odor activity value (OAV) for tamarillo. Relative OAVs were calculated by dividing the relative concentrations of VCs by their odor thresholds. For the compounds that had a range of odor thresholds quoted rather than a single number (e.g., 5-hydroxymethylfurfural), the lowest value of the odor threshold was applied to calculate the relative OAV. Only the compounds with relative OAVs greater than 1 were acknowledged to contribute to the tamarillo aroma. The odor description of these VCs was retrieved from Acree and Arn [16].

### 2.6. Preparation of Extracts for Antibacterial Activity Testing

In order to prepare the tamarillo extracts, extraction process according to Oladele, Blessing, and Okosodo [17] was carried out with modification. One gram of freeze-dried peel and pulp of tamarillo were extracted with 10 mL of four different solvents: MilliQ, *n*-hexane, ethanol, and methanol. A vortex Genie set at room temperature was used for extraction for 24 h. The mixtures were transferred to 50 mL tubes and centrifuged for 10 min at 4000 rpm at 4 °C. The supernatant was collected and stored at 4 °C until further analysis. The final concentration of each extract was 100 mg mL^−1^.

### 2.7. Evaluation of Antibacterial Activity

Two Gram-positive bacteria (*Streptococcus pyogenes* and *Staphylococcus aureus*) and two Gram-negative bacteria (*Escherichia coli* and *Pseudomonas aeruginosa*) were provided by Auckland University of Technology’s culture collection (cryobank −80 °C freezer). The strains were cultured on nutrient agar for *E. coli* and blood agar for the other three bacteria prior to experimental use, and then subcultivated to obtain pure cultures, followed by incubation for 24 h at 37 °C. These bacteria were then used as the inoculate to prepare liquid cultures for the determination of antibacterial activity. Turbidity of 0.5 McFarland standard in the nutrient broth medium was used with lawn culture method to subculture on blood agar plates. Sterile discs of 13 mm diameter were dipped in 115 µL of the prepared extracts (described in 2.6) and placed on plates to study the effect of extracts on bacterial growth. All plates were incubated for 18 to 24 h at 37 °C to obtain colonies. Water was used as a control and three antibiotics discs (MAST group Ltd., Bootle, United Kingdom)—amoxicillin (AUG30), penicillin (PG1C), and ciprofloxacin (CIP5C)—purchased from Fort Richards Laboratories were also used as positive controls. The plates were left at room temperature for 30 min to allow diffusion of materials in media. The plates were sealed with parafilm, labeled, and placed in an incubator set to 37 °C. After 24 h of incubation, each plate was examined for inhibition zones around the sterile disc wells and measured to the nearest 0.1 mm. The antibacterial activity was expressed as the diameter of inhibition zones produced by the extracts against the test bacteria.

### 2.8. Statistical Analysis

All of the analytes were measured in at least triplicate, and the results are presented as mean ± standard deviation. For comparison about volatile content among different tissues, one-way analysis of variance (ANOVA) was applied using SPSS 25.0 (IBM Corp., Armonk, New York, NY, USA). Two-way ANOVA was applied to identify significant differences between different solvent extracts and bacteria in the antibacterial experiment. Fisher’s (LSD) multiple comparison tests were used to differentiate the differences between the means. A *P* value < 0.05 was considered statistically significant.

## 3. Results and Discussion

### 3.1. Impact of Freeze-Drying in Volatile Components of Tamarillos

All of the volatiles identified in fresh pulp and peel were also detected in freeze-dried pulp and peel, respectively (Supplementary Materials Tables S1 and S2). There were 5 and 22 compounds that were detected in the freeze-dried form but absent in the fresh form for pulp and peel, respectively. These may possibly be present in trace amounts below the detection limit. The total number of VCs detected were 68 and 73 in fresh and freeze-dried pulp, respectively. There was a partial loss in the amounts of volatiles from the freeze-drying process. As outlined in Supplementary Table S1, 36 and 32 compounds were detected by SPME–GC–MS in either higher or lower amounts in the freeze-dried pulp compared to its fresh form, respectively, and a similar pattern was found for the peels (Supplementary Materials Table S2). Although in low concentration, methyleugenol, methyl salicylate, propyl-cyclopropane, and 1-methoxy-3-methyl-3-butene were present in higher magnitudes in the fresh pulp compared to the freeze-dried form. However, these compounds were not the major volatile constitutes of tamarillo and are unlikely to have an impact on the overall fruit aroma between the fresh and freeze-dried forms. By contrast, the freeze-dried samples contained significantly higher concentrations of ethyl hexanoate, ethyl butanoate, and nonanal. Concentrations of methyl butanoate, methyl hexanoate, methyl octanoate, and (Z)-3-hexen-1-ol were not different between fresh and freeze-dried tamarillo (*P* > 0.05). Ethyl butanoate, ethyl hexanoate, methyl butanoate, methyl hexanoate, methyl octanoate nonanal, and (Z)-3-hexen-1-ol have been previously reported as the major volatiles of fresh tamarillos (Table 1) [6,7,8].

**Figure 1:**
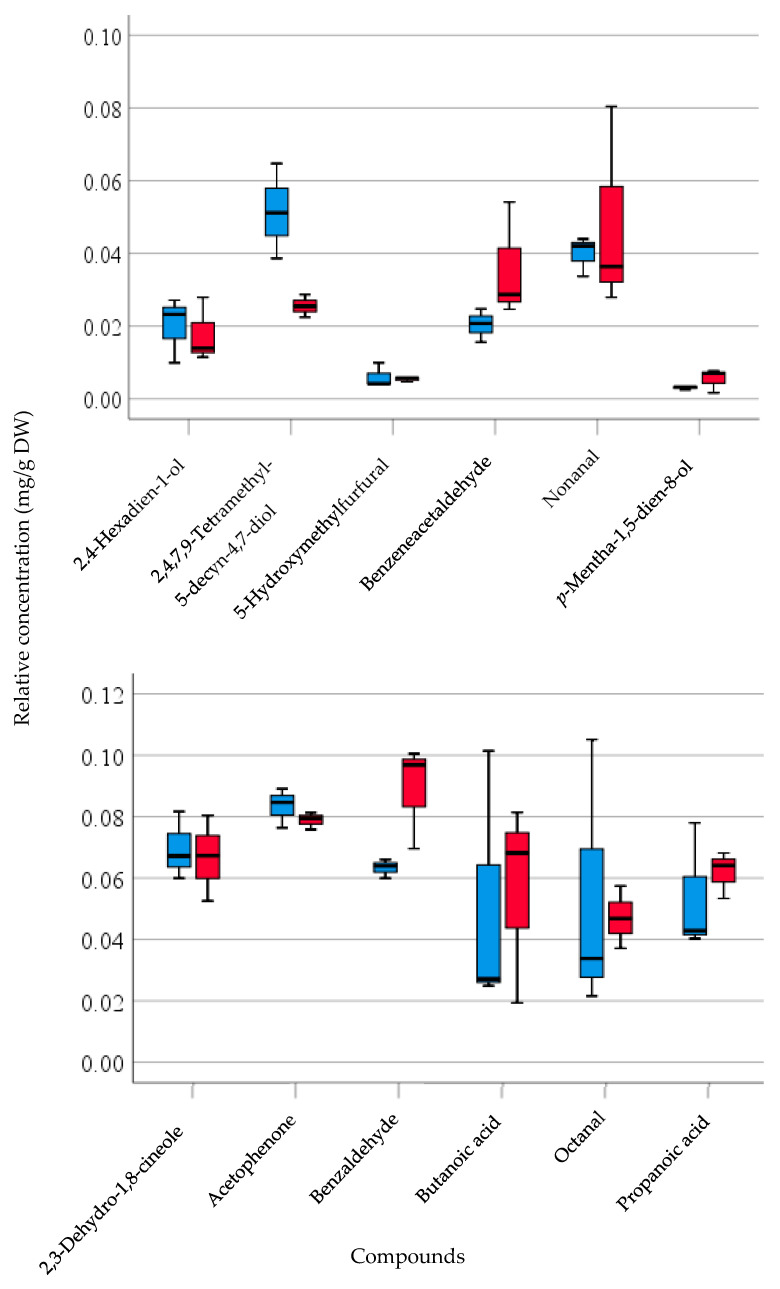
Comparison of relative concentration (mg/g DW) with respect to the internal standard of selected volatile compounds between fresh and freeze-dried pulp of tamarillo analyzed by TD–GC–MS. Data are presented showing median and quartiles in box-plot, and a range of each compound shown in whisker (*n* = 3). (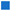 Fresh, 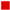 Freeze-dried sample).

Figure 1 shows that insignificant differences (*P* > 0.05) in low boiling point volatiles were found between fresh and freeze-dried samples when measured using TD–GC–MS. These include 2,4-hexadien-1-ol, 5-hydroxymethylfurfural, nonanal, 2,3-dehydro-1,8-cineole, acetophenone, butanoic acid, octanal, and propanoic acid. Difference in relative concentration of these volatiles between fresh and freeze-dried samples ranged from 4.12% to 17.27%, being the lowest and highest of 2,3-dehydro-1,8-cineole and nonanal, respectively. For 2,4,7,9-tetramethyl-5-decyn-4,7-diol, benzeneacetaldehyde, p-mentha-1,5-dien-8-ol, and benzaldehyde, the difference between fresh and freeze-dried samples was above 20%, in which higher relative concentration had been detected in the freeze-dried samples than in the fresh form (Figure 1). Results from both SPME and TD–GC–MS in the current study demonstrated that during the freeze-drying process, the main volatiles are retained.

### 3.2. Comparison between SPME and TD–GC–MS for Analyzing Volatiles

Identities were assigned to 121 features in the TD–GC–MS data, and these were quantified relative to the internal standard in peel and pulp of tamarillo. Table 2 lists these VCs in the order of chemical class, along with their retention index and mass-to-charge ratio (*m*/*z*). In the current study, we detected 117 and 73 volatiles in New Zealand red tamarillo pulp using TD–GC–MS and SPME–GC–MS, respectively. Compared to 46 VCs in Colombian red tamarillo [7], 49 volatiles in Malaysian red tamarillo [8], and 33 volatiles in Panama reddish-purple tamarillo from using pulp samples with solvent extraction and HS-SPME for analysis [6], the use of TD–GC–MS improved identification of volatiles with both higher sensitivity and a wider detection range. All of the furan, nitrogen-, and sulfur-containing compounds, benzenes, hydrocarbons, carboxylic acids and derivatives, as well as most fatty acids (17/19 compounds) volatiles detected by TD–GC–MS in the current study have never been reported in tamarillo by others who used SPME. This was confirmed from our data as well, with absence of some volatiles with low boiling points (benzeneacetaldehyde, acetophenone, octanal, and propanoic acid). The TD–GC–MS method required no preheating, in contrast to SPME, and did not alter the sample, enabling retainment of odoriferous properties of samples in the extraction process.

Solvent extraction (water, dichloromethane, and/or a mixture of pentane-dichloromethane) followed by drying to concentrate aroma compounds has been used to study volatiles in tamarillo [7,8,18]. Du and Qian [19] noted that dichloromethane had a poor elution power for some of the VCs, including furaneol, which contributes significantly to the aroma of tamarillo. As mentioned by Ruan, Aalhus, Juárez, and Sabik [20], liquid extraction could lead to loss and degradation of analytes. This problem was partially resolved with the use of TD in this study. Improvement in analytical sensitivity with the use of TD by enhanced desorption efficiency and sample dilution [21] reduced manual sample preparation time and minimized interference from impurities in organic solvent seen in GC analysis [20]. According to Yang, Zhang, Yin, Deng, Jiang, Yuan, Dong, Li, Hua, and Wang [22], TD can be used to detect volatile and semi-VCs in a diversity of sample types. As aroma compounds are directly released from the adsorbent under high temperatures and these are concentrated by cryofocusing prior to separation by GC [22], TD may be superior in volatile analysis compared to the traditionally used HS-SPME as suggested by Kücklich, Möller, Marcillo, Einspanier, Weiß, Birkemeyer, and Widdig [23]. It was confirmed in the current study that the combination of TD–GC–MS provides a simple, quick, and comprehensive aroma analysis of tamarillo compared with SPME.

### 3.3. Volatile Compounds in Peel and Pulp of Freeze-Dried Tamarillos

**Figure 2:**
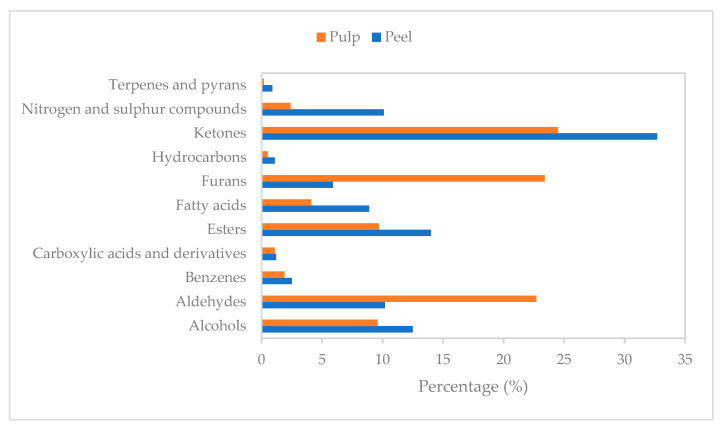
Relative percentage contents (%) of chemical groups identified in the pulp and peel of tamarillo.

Fruit volatiles consist of compounds from various chemical groups (alcohols, aldehydes, esters, ketones, furanones, and terpenes), which are formed from precursors as the fruit ripens and changes postharvest [24]. For example, sucrose content is associated with the production of furans, whereas fatty acids content is related to the synthesis of esters and alcohols through lipid degradation [25]. Among a total of 121 features, peel had two less VCs than pulp (115 compounds compared to 117). The VCs were further classified to their chemical groups based on chemical structure: 20 esters, 20 ketones, 19 fatty acids, 11 nitrogen compounds, 10 aldehydes, 10 furans, 8 alcohols, 8 benzenes, 6 hydrocarbons, 4 carboxylic acids and derivatives, 2 sulfur compounds, 2 terpenes, and 1 pyran compound. Although the concentrations of chemical groups and VCs were tissue-dependent, the largest proportion in both tissues of tamarillo was comprised of ketones with 32.7% and 24.5% for peel and pulp, respectively (Figure 2). Ketones are mainly derived from lipid oxidation as well as from citrate and glucose metabolism [25]. They normally contribute to fruity notes with 4-hydroxy-4-methyl-2-pentanone and 3,5-dihydroxy-2-methyl-4H-pyran-4-one being the most abundant ketone in peel and pulp samples, respectively (Table 2). Some ketone volatiles are important contributors to tamarillo aroma and flavor [6,7,8,18]. Pulp had a higher concentration of maltol and a derivative of maltol (3,5-dihydroxy-2-methyl-4H-pyran-4-one or 5-hydroxymaltol) than peel. These compounds may serve as flavor enhancers, flavoring agents, or fruit flavor additives. Maltol is known to enhance oral bioavailability of gallium- [26] as well as iron-based drugs [27], which adds to the potential of using freeze-dried tamarillo as a therapeutic adjunct.

Esters, with relative percentage contents of 13.7–20.1% and 9.7–11.7% in peel and pulp, respectively, also contribute to the tamarillo aroma. The most common aroma notes of ester compounds are fruity or floral [25]. Esters were the second dominant volatile group in tamarillo peel (Figure 2). Phthalic acid, hept-4-yl isobutyl ester, and 3-furancarboxylic acid, methyl ester were the abundant ester compounds in peel and pulp, respectively (Table 2). The contribution of ethyl butanoate, ethyl hexanoate, methyl butanoate, methyl hexanoate, and methyl octanoate as key esters of tamarillo aroma has been reported previously as summarized in Table 1. Some of these volatiles have been found in both golden-yellow and reddish-purple tamarillos from Panama with relative amounts of 5.4–5.9% for ethyl hexanoate, 1.6–2.7% for ethyl butanoate and 0.7–1.0% for ethyl hexadecanoate [6]. These esters have also contributed to the volatile profile of New Zealand tamarillo (Table 2). Ethyl hexanoate is used in perfumes and as fruit flavors. Ethyl butanoate is used as a flavor enhancer in processed orange juices, while ethyl hexadecanoate carries a waxlike aroma [28].

According to Wong and Wong [8], alcohol compounds are formed through enzymatical reactions from unsaturated acids during the maceration of tamarillo. Flavor volatiles of New Zealand tamarillo were dominated by eight different types of alcohols with 2,3-butanediol as the most abundant (Table 2). This volatile was also present in Colombian tamarillo with relative amount of <100 μg/kg FW [7,18] and was also found as one of dominating alcohol in other fruit, such as *Taxus baccata* L. Red Arils [24]. Eugenol and α-terpineol, the major volatiles in tamarillo from Colombia [7,18], Malaysia [8], and Panama [6], were identified in the current study. Eugenol carries sweet and phenolic flavor [29] and used in perfumes, flavorings, and essential oils [30]. α-terpineol has pleasant odor and it is a commonly used ingredient in perfumes, cosmetics, and flavors [31].

A total of 10 aldehyde compounds were detected in freeze-dried tamarillo. With a fruity or floral aroma, most aldehydes contribute to fresh note of fruit [25]. Among this class, nonanal was one of the abundant volatiles, which was also seen in reddish-purple tamarillo from Panama with a relative amount of 9.0% [6]. Nonanal carries fatty-floral and citrus flavor [32] and possesses fungicidal effect toward *Penicillium cyclopium* with minimum inhibitory concentration and minimum fungicidal concentration of 0.3 and 0.4 mL L^−1^, respectively [33]. Nonanal was also detected in both peel and pulp of red tamarillo sourced from New Zealand (Table 2).

This was the first study to detect 10 compounds of the furan group in tamarillo with furaneol being the dominant compound. Synthesis of furan compounds is related to the sucrose content in fruit [25]. Furans usually contribute to caramel-like, sweet, and fruity notes of fruit. Furaneol is one of significant volatiles in tomato, mango, raspberry, pineapple, and strawberry and it is used as a flavoring agent [34] that gives the “caramel-like”, “strawberry-like”, “burnt-pineapple”, and “sweet, floral” flavors [19]. Another abundant furan compound, 5-hydroxymethylfurfural, might have been released due to the thermal breakdown induced by the TD process as it is a common artefact formed from hexose sugars by Maillard reaction during heat-treatment of foods [35]. This compound is also present in tomato and is used as an index of heat treatment and deterioration in tomato paste, fruit juice, honey, and other foods. In the food industry, 5-hydroxymethylfurfural is also used as a food additive and flavoring agent [36].

Apart from the five dominant groups, fatty acids have partially contributed to the volatile profile of freeze-dried tamarillo. According to Belitz, Grosch, and Schieberle [37], autoxidation of unsaturated lipids in fruits may produce fatty acids, and this process is enhanced with heat treatment. Though the TD process, the lipids may have been broken down into free fatty acids, although the use of helium gas may have not significantly induced the autoxidation. Among 19 fatty acids that were identified in tamarillo, only butanoic acid and hexanoic acid with relative amount of <100 μg/kg FW was previously reported in tamarillo from Colombia [7].

All of the nitrogen- and sulfur-containing compounds, benzenes, hydrocarbons, pyrans, and terpenes, as well as carboxylic acids and derivatives, also contributed to tamarillo volatiles (Table 2). Nitrogen- and sulfur-containing compounds are associated with green notes of fruit [25]. Sulfur-containing compounds are produced from the synthesis and degradation of sulfur amino acids such as methionine and cysteine. Presence of benzoic acid in tamarillo indicates that it may possess potential as a natural antifungal agent [38]. Dimethyl sulfone (sulfur-containing compound) with known anti-inflammatory effects [39] was also found. Farnesol or 3,7,11-trimethyl-2,6,10-dodecatrien-1-ol, a terpene compound, carrying sweet floral flavor notes [25], was also detected, which has been used as a chemopreventative and antitumor agent [40] and has shown antibacterial activity [41]. However, one of the nitrogen-containing compounds, docosenamide or erucamide, is a common contaminant from the use of polypropylene lab materials [42], which may have come from Eppendorf tube for storing the samples in this study. Because this is an endogenous metabolite, further analysis may be required to determine whether it is actually present as a VC in tamarillo or not.

This is the first study comparing volatile component in peel and pulp of tamarillo. The volatile profile in peel tissue and its contribution to total aroma of tamarillo remain scarce. Data from the current study showed that peels contained as much as pulp of the main volatiles. Although the peel is often discarded as a byproduct, it has a potential to be used as an aroma enhancer in freeze-dried form.

### 3.4. Odor Threshold and Relative Odor Activity Value (OAV) of Freeze-Dried Tamarillo

OAV, known as a ratio of concentration to odor threshold of the volatile, indicates contribution of each compound to the typical flavor of any food type [43]. A VC significantly contributes to the overall fruit odor if its OAV is greater than 1—the higher the OAV, the greater the possibility of a VC to be perceived [43]. The odor detection threshold in water (ppb) and the relative OAVs for several VCs found in the peel and pulp of freeze-dried tamarillo are listed in Table 3. Due to a lack of odor threshold data in the literature, a total of 36 compounds from 121 detected volatiles are presented with the relative OAVs. Of these, 13 and 10 of these volatiles were present beyond their odor threshold for pulp and peel, respectively. These include 5 aldehydes, 2 alcohols, 2 esters, 2 furans, and 1 hydrocarbon.

Five and three aldehyde compounds were found in relative amounts higher than their threshold concentrations (relative OAVs > 1) for pulp and peel, respectively. Methional, perceived as potato skin and tomato, [44], had the highest relative OAV in all of the analyzed samples. The relative OAV of methional in pulp was significantly higher than that in peel by approximately 7.5 times. By contrast, the relative OAV of nonanal and (E)-2-decenal for peel was higher than that for pulp, by approximately 1.6 and 1.8 times, respectively. These two compounds also significantly contributed to the overall aroma of peel and pulp red tamarillo. Nonanal is associated with fatty, green, and citrus odor, while (E)-2-decenal has fatty and green notes. With the relative OAVs greater than 1, octanal is considered as an important aroma contributor to the overall flavor of tamarillo pulp. This volatile is characterized by fatty, lemon, and green notes. Only 3-furaldehyde was found in pulp to have relative OAV > 1 (Table 3).

Two alcohols were present in relative contents higher than their odor detection thresholds. The relative OAVs of 2,3-butanediol for pulp was higher than that for peel. This indicates that a greater contribution to the overall flavor of tamarillo comes from the pulp rather than the peel for 2,3-butanediol, which is characterized by fruity and creamy notes (Table 3).

Two esters were identified as present in higher concentration than their threshold values. It is interesting to note that hexanoic acid, ethyl ester, and ethyl hexanoate have greater contributions to the overall flavor of pulp rather than peel. This ester compound is associated with sweet, fruity, and apple peel-like notes. By contrast, propanoic acid, 2-methyl-, ethyl ester, owning a sweet and fruity odor, is likely to be more involved to the characteristic flavor of peel.

Pulp and peel volatiles of tamarillo had fruity, caramel, and burnt pineapple-like odors, which are characteristics of furaneol. Furaneol is one of the most important aroma compounds in tomato [19] and, from our results, for tamarillo as well. The relative OAV of furaneol for pulp was higher than that of peel by approximately 2.5 times. Meanwhile, 5-hydroxymethylfurfural, carrying caramel and buttery odor, had the relative OAV greater than 1 in pulp, only (Table 3).

One hydrocarbon compound, 1,1,5-trimethyl-1,2-dihydronaphthalene, has significantly contributed to provide the distinctive flavor of tamarillo with the relative OAV of greater than 1. This compound contributed more toward the peel rather than the pulp and it is characterized by a licoricey note and wine odor. Ketones were the dominant volatiles in tamarillo; however, only one ketone compound, 4-hydroxy-4-methyl-2-pentanone, contributed toward the flavors of both peel and pulp of tamarillo when assessed using the relative OAV. Again, this ketone compound, having a minty note, was less involved to the characteristic flavor of pulp rather than peel (Table 3).

Fatty acids and nitrogen-containing compounds in tamarillo were below the odor detection thresholds. Not all of the detected VCs from this study had known odor detection thresholds in the literature; therefore, further investigation may be needed. Some of the identified volatiles having relative OAVs of less than 1 may possibly have contributed to the fruity characteristic of tamarillo synergistically. Hence, quantitative analysis using dynamic head space coupled with GC–MS and a sensory trial with trained panelists would help to advance the understanding of how these VCs contribute to the overall perception of tamarillo flavor.

Overall, methional was the most significant contributor to the flavor of both peel and pulp of tamarillo, followed by 2,3-butanediol. Apart from these two compounds, nonanal highly contributed to the flavors of peel, whereas the major contributor to the characteristic flavor of pulp was octanal.

### 3.5. Antibacterial Activities of Different Solvent Extracts from Tamarillo

For the assessment of antibacterial activities of tamarillo extracts, control tests were performed against two bacteria which are known to be present in human gastrointestinal tract and have an easier path to infecting an individual. Bacteria were inoculated on blood agar plate and later streaked on Mueller Hinton (MH) agar plates. Growth of *E. coli* on MH agar plates with ciprofloxacin and penicillin as the antibiotics is presented in Figure S1A. The strain did not lose its pathogenicity and the antibiotics used were effective. This assured the ability to perform further experimentation using the same culture. Penicillin disc had no effect on the growth of *E. coli* and was not used for further testing of antibacterial activity.

Figure S1B shows that for *E. coli*, the amoxicillin and ciprofloxacin discs exhibited antibacterial activity with zone of inhibition of 11 to 13 mm and 32 to 34 mm in diameter, respectively. *E. coli* was only resistant to penicillin as observed earlier in Figure S1A. For *Pseudomonas aeruginosa*, the antibiotic activity was shown only from ciprofloxacin disc, with zone of inhibition ranging from 18 to 22 mm in diameter. It showed resistance to both amoxicillin and penicillin (Figure S1C). This could be due to the evolution and increasing use of penicillin over time against the same bacterial strain or culture, making it resistant to penicillin. From these readings, it was confirmed that bacterial strains were appropriate to be tested for antibacterial activity of tamarillo extracts.

**Figure 3:**
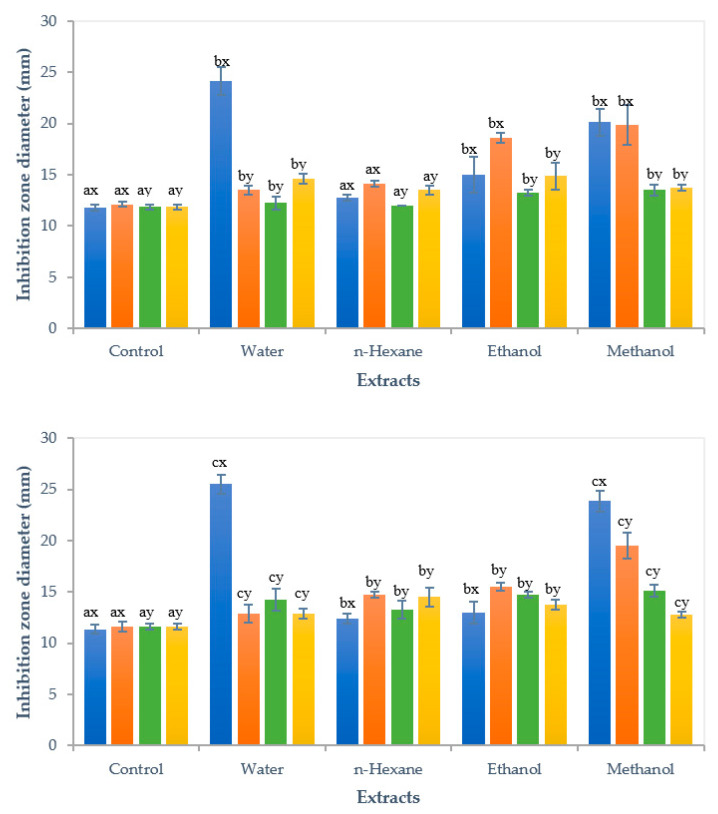
Antibacterial activity of peel (top) and pulp (bottom) extracts of red tamarillo. Data are presented as mean (mm) and standard deviation in error bar (*n* = 3). Different letters indicate statistical difference (*P* < 0.05) using Two-way ANOVA. Means shown in a, b, c are significantly different at *P* < 0.05 between solvent extracts. Means shown in x and y are significantly different at *P* < 0.05 between bacteria. (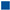 *Escherichia coli*, 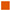 *Pseudomonas aeruginosa*, 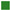 *Staphylococcus aureus*, 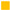 *Streptococcus pyogenes*). Control was water without any tamarillo extract.

All extracts from tamarillo peel and pulp exhibited inhibitory activity against all test bacteria with significant impact (*P* < 0.05), as shown in Figure 3. For the peel, inhibitory effect of water extract on *E. coli* was the greatest, followed by the methanol extract on the same bacteria with the mean inhibition zone diameter of 24.13 and 20.13 mm, respectively. The greatest inhibitory effect of water extracts on *E. coli* had been reported from orange, yellow lemon and banana peels compared to other extracts [45]. The methanol extracts of tamarillo peel showed significantly higher antibacterial activity than other peel extracts on *P. aeruginosa* and *S. aureus*, whereas the highest inhibition zones on *S. pyogenes* was observed for ethanol extract. The methanol extract of tamarillo peel showed better inhibitory effects on *E. coli* compared to methanol extract of feijoa peel at concentration of 100 mg mL^−1^ with inhibition zone of 14.7 mm [46]. It was also observed that water, ethanol, and methanol extracts from tamarillo peel were approximately 20%–47% more effective on Gram-negative bacteria as compared to Gram-positive bacteria (Figure S2). This could be attributed to the differences in the cell wall structures of these bacteria. The Gram-positive bacteria have a thick peptidoglycan cell wall in multilayer, which acts as an obstacle to various environmental materials, including natural as well as synthetic antibiotics. By contrast, the Gram-negative bacterial cell has a single peptidoglycan outer layer, which is a less effective penetrability barrier, and also does not contain teichoic acid, which is present in Gram-positive bacteria [47]. Furthermore, Gram-negative bacteria have low resistance to physical disruption due to a weak cell wall structure [45]. Presence of phenolic compounds and flavonoids in the extracts might be responsible for excellent antimicrobial activities of tamarillo peel, as similar results had been observed in pomegranate peels [48].

For tamarillo pulp, the average inhibition zone of water extract was also the highest at 25.5 mm for *E. coli*. Methanol extract showed the greatest inhibitory activity against *P. aeruginosa* and *S. aureus* with the diameter of 19.5 and 15.1 mm, respectively, which were similar to the effects seen with the peel extracts. Methanol extract of tamarillo fruit showed better antimicrobial activity against *E. coli* and *S. aureus* than that of pomegranate fruit (1000 mg mL^−1^) with the inhibition zone diameter of 12 and 22 mm, respectively [49]. Differentiated from the peel extract, hexane extract from the pulp had the highest inhibition zone diameter for *S. pyogenes*. It was also observed that water and methanol extracts from both peel and pulp showed the greatest inhibition activity on *E. coli,* whereas hexane and ethanol extracts from both tissues had the highest inhibition zone diameter for *P. aeruginosa* when comparing to other pathogens. At the same concentration, water extracts of both tamarillo peel and pulp showed greater effect on inhibition of *E. coli* growth than that of peel and pulp of feijoa which failed to show any activity against this pathogen [46]. Water and methanol extracts from tamarillo pulp showed over 40% more effective on Gram-negative bacteria compared to Gram-positive bacteria (Figure S3).

The greater antimicrobial efficacy on *P. aeruginosa* and *S. pyogenes* was observed from the water, methanol and ethanol extracts of tamarillo peel rather than the pulp tissue. In contrast, all pulp extracts showed better inhibitory effect on *S. aureus* compared to the peel extracts. For *E. coli*, water and methanol extracts of the pulp showed greater antimicrobial activity, whereas the ethanol and hexane extracts of pulp exhibited opposite activity in comparison with peel extracts. The differences might be due to different components in peel and pulp extracts. Higher concentration of vitamin C, kaempferol-3-rutinoside, delphinidin rutinoside, and pelargonidin rutinoside in pulp than in peel of Laird’s Large tamarillo [1,3] had been observed, which might explain better of the inhibitory effect of pulp extracts on *S. aureus* (for all extracts) and *E. coli* (for water and methanol extracts). Peel had higher concentrations of α-tocopherol, chlorogenic acid, caffeic acid, rutin, isorhamnetin rutinoside, and cyanidin rutinoside than pulp [1,3], showing greater inhibitory effect of peel extracts on *P. aeruginosa* (for water, methanol and ethanol extracts), *S. pyogenes* (for water, methanol and ethanol extracts), and *E. coli* (for hexane and ethanol extracts).

Tamarillo is a good natural source of vitamins, bioactives (phenolics and anthocyanins), and some amino acids (glutamic acid, aspartic acid, and GABA) from our previous work (Diep et al., 2020 a, b and c). All of these results confirm that freeze-dried tamarillo has a great potential to be utilized as a natural additive to enhance aromas and flavors and shelf life of food product.

## 4. Conclusions

For the first time, the VC and antibacterial activities of Laird’s Large and red tamarillo from New Zealand were evaluated. Freeze-drying was able to preserve most of the VCs found in fresh fruit. With the use of TD–GC–MS, 82 new VCs were detected. The major VCs present in the tamarillo peel were 4-hydroxy-4-methyl-2-Pentanone; 3,5-dihydroxy-2-methyl-4H-Pyran-4-one, and Phthalic acid, hept-4-yl isobutyl ester with 912, 609, and 562 μg/g DW, respectively, whereas 5-Hydroxymethylfurfural (5796 μg/g DW), 3-Furaldehyde (4313 μg/g DW), and 3,5-dihydroxy-2-methyl-4H-Pyran-4-one (2767 μg/g DW) were the major abundant volatiles for the pulp tissue. However, from SPME–GC–MS data, the most abundant VC in both fresh and freeze-dried tamarillo was hexanoic acid methyl ester for pulp (30 and 37%), and (E)-3-Hexen-1-ol for peel (36 and 29%), respectively. The major contributor to the overall flavor of tamarillo was methional, which is associated with tomato-like flavor notes. This may help explain the original name of tree tomato. Further studies including smell and taste threshold and identification tests and quantification of VCs will be able to advance the sensorial characterization of tamarillo composition, flavor, and odor, which may suggest new uses of this fruit. Preliminary evidence is presented for antibacterial activity in both peel and pulp tissue extracts of tamarillo. Water extract from peel and pulp showed the greatest inhibition of *E. coli* (minimum 24 mm inhibition zone), whereas the methanol extract of both tissues showed greatest inhibitory activity against *P. aeruginosa* (approximately 19.5 mm inhibition zone) and *S. aureus* (minimum 13.5 mm inhibition zone). Water and methanol extracts from both peel and pulp were more effective on Gram-negative bacteria than on Gram-positive bacteria. The inhibitory effect of water extract on growth of the bacteria tested is of particular interest for application to the food industry. As highlighted, the peel, which are often discarded as by-product, has a great potential to be used as a natural preservative to enhance flavor and shelf life of other food products in freeze-dried form. Utilization of the peel will help to reduce food waste and contribute positively toward the economy and environment.

## Figures and Tables

**Table 1:** Relative percentage of major volatile compounds in tamarillo pulp from this study compared to other studies.

Volatile/References	Methyl butanoate	Methyl hexanoate	Methyl octanoate	(Z)-3-hexen-1-ol	Ethyl butanoate	Ethyl hexanoate	Nonanal
This study	8.5%	36.9%	0.2%	1.6%	0.55%	0.51%	0.27%
Durant, et al. [6]	n.d.	4.6%	n.d.	n.d.	2.7%	5.4%	9.0%
Torrado, et al. [7]	<100 μg/kg	>500 μg/kg	n.d.	>500 μg/kg	n.d.	<100 μg/kg	n.d.
Wong and Wong [8]	n.d.	8.6%	0.2%	26.6%	n.d.	1.0%	n.d.

n.d.: not detected.

**Table 2:** Volatile compounds and their relative contents in peel and pulp of freeze-dried tamarillo are presented as mean ± SD (*n* = 3) and listed in the order of chemical groups and then retention index. Letters (a, b) indicate statistical difference (*P* < 0.05) across each row.

No	Compounds	RI	*m*/*z*	Relative Concentration (μg/g DW) to the Internal Standard	
				Peel	Pulp
*Alcohols*					
1	R-(-)-1,2-Propanediol	975.9	45.1	287 ± 64.3 ^a^	1062 ± 100 ^b^
2	2,3-Butanediol	996.6	45.1	492 ± 319 ^a^	1692 ± 1241 ^b^
3	Alpha-terpineol	1285.7	59.1	0.1 ± 0.1 ^a^	0.7 ± 0.6 ^a^
4	*p*-Mentha-1(7), 2-dien-8-ol	1487.6	94	0.7 ± 0.1 ^a^	1 ± 0.2 ^a^
5	Eugenol	1536.1	164.1	59.6 ± 34.9 ^a^	10.7 ± 5.4 ^b^
6	2,4,7,9-Tetramethyl-5-decyn-4,7-diol	1571.0	151.1	14.5 ± 2 ^a^	25 ± 7.7 ^b^
7	trans-Isoeugenol	1614.9	164	40.2 ± 28.6 ^a^	5.6 ± 3.2 ^b^
8	2,6-Dimethoxy-4-(2-propenyl)-phenol	1808.5	194	37.9 ± 26.8 ^a^	9 ± 3.7 ^b^
*Aldehydes*					
9	3-Furaldehyde	977.3	95	484 ± 161 ^a^	4313 ± 1195 ^b^
10	Methional	1036.4	104	15.4 ± 4.2 ^a^	118 ± 8.1 ^b^
11	Octanal	1078.4	55.1	n.d.	36.6 ± 1.6
12	5-Methyl-2-furancarboxaldehyde	1096.2	110.1	102 ± 26.4 ^a^	1913 ± 104 ^b^
13	Nonanal	1176.8	57	19.2 ± 16 ^a^	12 ± 3.4 ^b^
14	1H-Pyrrole-2-carboxaldehyde	1209.7	94	10.9 ± 12.2 ^a^	29.6 ± 3.7 ^b^
15	(E)-2-Decenal	1364.9	70.1	2.3 ± 1.4 ^a^	1.2 ± 0.1 ^a^
16	2-Undecenal	1472.5	70.1	n.d.	0.8 ± 1.4
17	4-Methyl-benzaldehyde	1479.4	120	4.7 ± 1 ^a^	7.5 ± 0.3 ^b^
18	2,4-Dihydroxy-6-methyl-benzaldehyde	1637.8	151	0.6 ± 0.6 ^a^	7.1 ± 0.8 ^b^
*Benzenes*					
19	4-Ethenyl-1,2-dimethyl-benzene	1138.6	132	7.3 ± 2.9 ^a^	47.2 ± 2.6 ^b^
20	Benzeneacetaldehyde	1163.8	91.1	114 ± 32.9 ^a^	405 ± 11 ^b^
21	2-Acetoxy-5-hydroxyacetophenone	1429.6	137	8.6 ± 3.3 ^a^	21.3 ± 0.7 ^b^
22	3′,5′-Dihydroxyacetophenone	1429.8	137.1	8.6 ± 3.3 ^a^	21.4 ± 0.8 ^b^
23	2′,6′-Dihydroxy-3′-methylacetophenone	1514.1	151	5.6 ± 1.4 ^a^	15.9 ± 0.3 ^b^
24	1-Ethenyl-4-(2-methylpropyl)-benzene	1603.2	117	n.d.	n.d.
25	(E)-2,6-Dimethoxy-4-(prop-1-en-1-yl) phenol	1922.4	194	9.9 ± 7.5 ^a^	3.6 ± 1 ^a^
26	Diphenylacetylene	1956.5	178	2.7 ± 1.7 ^a^	3.7 ± 0.1 ^a^
*Carboxylic acids and derivatives*					
27	Methylene-cyclopropane carboxylic acid	1028.7	98.1	5.9 ± 8.4 ^a^	81 ± 23.3 ^b^
28	Benzoic acid	1403.9	105	17.2 ± 5.5 ^a^	71.3 ± 0.9 ^a^
29	Valeric anhydride	1532.4	85	27.1 ± 4.8 ^a^	110 ± 87.8 ^b^
30	3-Amino-4-hydroxybenzoic acid	1678.7	153	22.3 ± 3.8 ^a^	41.2 ± 1 ^b^
*Esters*					
31	Hexanoic acid, ethyl ester	1055.4	88	5.6 ± 2.4 ^a^	11.8 ± 2.7 ^a^
32	Butanoic acid, 3-hydroxy-, ethyl ester	1065.7	88.1	5.6 ± 2.4 ^a^	5.6 ± 8 ^a^
33	2-Propenoic acid, 2-methyl-, (tetrahydro-2-furanyl) methyl ester	1113.9	71.1	8.8 ± 2.8 ^a^	77.4 ± 7.6 ^b^
34	Propanoic acid, 2-methyl-, ethyl ester	1238.5	71.1	33.2 ± 13.3 ^a^	29.9 ± 3.3 ^a^
35	3-Furancarboxylic acid, methyl ester	1243.6	95	79.8 ± 20.7 ^a^	1585 ± 161 ^b^
36	Aspirin methyl ester	1289.4	120.1	1.7 ± 0.3 ^a^	1.2 ± 0.1 ^a^
37	1,2,3-Propanetriol, 1-acetate	1440.7	61	18.4 ± 15.6 ^a^	28.7 ± 2.9 ^a^
38	Propanoic acid, 2-methyl-, 3-hydroxy-2,2,4-trimethylpentyl ester	1505.3	71.1	8.9 ± 12.6 ^a^	10 ± 2.7 ^a^
39	Glycerol 1,2-diacetate	1527.6	43.1	5.9 ± 3.4 ^a^	11.3 ± 13.7 ^a^
40	Tributyl phosphate	1667.2	99.1	57.7 ± 5.3 ^a^	89.1 ± 8.7 ^b^
41	Carbamic acid, methylphenyl-, ethyl ester	1905.0	179	27.5 ± 4.8 ^a^	121 ± 6.2 ^b^
42	2-Ethylhexyl salicylate	1928.0	120	1.5 ± 0.7 ^a^	1.3 ± 0.1 ^a^
43	Homosalate	2020.9	138	n.d.	0.1 ± 0
44	Phthalic acid, hept-4-yl isobutyl ester	2058.3	149	562 ± 238 ^a^	730 ± 12.9 ^b^
45	Hexadecanoic acid, ethyl ester	2073.5	88	34.8 ± 29.3 ^a^	7.3 ± 1.4 ^b^
46	Benzoic acid, 2-benzoyl-, methyl ester	2193.4	163	2.5 ± 0.8 ^a^	2.5 ± 1.1 ^a^
47	Ethyl oleate	2259.7	55.1	4.2 ± 0.2 ^a^	1.5 ± 0.2 ^b^
48	Hexanedioic acid, bis(2-ethylhexyl) ester	2544.5	129	6 ± 2 ^a^	5.2 ± 2.2 ^a^
49	1,2-Cyclohexanedicarboxylic acid, dinonyl ester	2924.2	155	3.1 ± 4.3 ^a^	0.5 ± 0.5 ^b^
50	Phthalic acid, nonyl 4-octyl ester	2981.9	149	2.4 ± 2.4 ^a^	2.2 ± 2.1 ^a^
*Fatty acids*					
51	Propanoic acid	947.5	74.1	25.1 ± 7 ^a^	31.3 ± 10.3 ^a^
52	Butanoic acid	1003.8	60	5.7 ± 5.3 ^a^	0.7 ± 0.6 ^b^
53	Hexanoic acid	1166.3	45.1	79.9 ± 34.8 ^a^	128 ± 8.8 ^b^
54	Heptanoic acid	1253.7	60	15.3 ± 7.2 ^a^	28.5 ± 0.5 ^b^
55	2-Ethyl-hexoic acid	1281.6	88	16.1 ± 6.8 ^a^	26.6 ± 1.5 ^b^
56	Octanoic acid	1353.4	60	47.6 ± 15.6 ^a^	86.5 ± 5.7 ^b^
57	Nonanoic acid	1448.0	60	62.9 ± 27.6 ^a^	97.7 ± 13.2 ^a^
58	n-Decanoic acid	1546.7	73.1	43.3 ± 18 ^a^	63.7 ± 10.5 ^a^
59	Dodecanoic acid	1749.4	73.1	119 ± 78.2 ^a^	261 ± 43.1 ^a^
60	Tetradecanoic acid	1932.9	73.1	n.d.	n.d.
61	Myristoleic acid	1944.2	69	8.7 ± 1.6 ^a^	16.6 ± 6.9 ^b^
62	Z-11-Tetradecenoic acid	1944.2	69.1	8.7 ± 1.6 ^a^	16.6 ± 6.9 ^b^
63	Pentadecanoic acid	2052.0	73	18 ± 0.6 ^a^	35.6 ± 9.7 ^b^
64	Palmitoleic acid	2144.5	69.1	15.4 ± 0.7 ^a^	28 ± 7.1 ^b^
65	n-Hexadecanoic acid	2155.4	73	94.7 ± 36.6 ^a^	234 ± 50 ^b^
66	9-Octadecenoic acid	2341.8	83.1	1.9 ± 1.4 ^a^	9.9 ± 7.3 ^b^
67	(E)-9-Octadecenoic acid	2341.8	69	2 ± 1.5 ^a^	13.3 ± 7.7 ^b^
68	(Z,Z)-9,12-Octadecadienoic acid	2347.9	81.1	0.9 ± 0.7 ^a^	27.3 ± 27.2 ^b^
69	Octadecanoic acid	2353.7	73	9.3 ± 6.6 ^a^	55 ± 8 ^b^
*Furans*					
70	2,4-Dihydroxy-2,5-dimethyl-3(2H)-furan-3-one	1065.4	101.1	5.7 ± 0.3 ^a^	52.1 ± 14.6 ^b^
71	Dihydro-3-methylene-2(3H)-Furanone	1075.2	98.1	11.4 ± 10.1 ^a^	47.7 ± 41.8 ^b^
72	3-Furancarboxylic acid	1097.3	112	28.1 ± 20 ^a^	81.6 ± 14.2 ^b^
73	.+/−.-Tetrahydro-3-furanmethanol	1113.4	71	8.6 ± 2.8 ^a^	77.3 ± 7.6 ^b^
74	Furaneol	1239.5	128	164 ± 36.8 ^a^	411 ± 17.5 ^b^
75	1-(2-furanylmethyl)-1H-pyrrole	1298.9	81.1	6.5 ± 1.9 ^a^	7.1 ± 0.3 ^a^
76	5-Acetoxymethyl-2-furaldehyde	1483.1	126.1	1.5 ± 0.2 ^a^	57.1 ± 4.5 ^b^
77	5-Hydroxymethylfurfural	1524.5	97	115 ± 18.6 ^a^	5796 ± 371 ^b^
78	Dihydro-4-hydroxy-2(3H)-furanone	1556.1	44.1	13.3 ± 4.8 ^a^	33.8 ± 25.7 ^a^
79	2,3,5-Trimethyl-1H-indole	1692.1	158.1	4.9 ± 3.1 ^a^	2.7 ± 2.2 ^a^
*Hydrocarbons*					
80	1,1,5-Trimethyl-1,2-dihydronaphthalene	1412.9	157.1	4.3 ± 1.5 ^a^	2.9 ± 0.1 ^a^
81	Fluoranthene	2301.0	202	0.6 ± 0.3	n.d.
82	Tricosane	2307.3	57	10.3 ± 6.1 ^a^	2.2 ± 0.7 ^b^
83	Hexadecanamide	2445.2	59.1	26.3 ± 23.3 ^a^	46.9 ± 24.1 ^a^
84	(Z)-9-Octadecenamide	2636.4	59.1	13.8 ± 11.4 ^a^	20.5 ± 11.8 ^a^
85	Octadecanamide	2649.2	59.1	26.5 ± 19.9 ^a^	61.3 ± 38.3 ^b^
*Ketones*					
86	4-Hydroxy-4-methyl-2-pentanone	992.4	59	912 ± 582 ^a^	617 ± 566 ^a^
87	1-(Acetyloxy)-2-propanone	1034.6	86.1	22.3 ± 24.7 ^a^	29.4 ± 13.6 ^a^
88	4-Cyclopentene-1,3-dione	1046.0	96	27 ± 8.8 ^a^	142 ± 7.6 ^a^
89	1-(4-Methylphenyl)-1-pentanone	1062.3	119	5.1 ± 2 ^a^	11.7 ± 6.3 ^b^
90	Butyrolactone	1118.9	86.1	42.2 ± 13.2 ^a^	154 ± 37.6 ^b^
91	2-Hydroxy-3-methyl-2-cyclopenten-1-one	1173.0	112	35.9 ± 14.8 ^a^	110 ± 8.9 ^b^
92	Acetophenone	1182.4	105	1.3 ± 1.1 ^a^	2.5 ± 0.1 ^b^
93	Phorone	1189.4	123	2.5 ± 3.6 ^a^	3.4 ± 0.2 ^a^
94	1-Methyl-2,4-Imidazolidinedione	1239.2	114	112 ± 22.8 ^a^	234 ± 10.5 ^b^
95	Furyl hydroxymethyl ketone	1243.9	95	79.7 ± 20.7 ^a^	1585 ± 161 ^a^
96	Maltol	1263.0	126.1	201 ± 53.4 ^a^	508 ± 24.9 ^b^
97	5-Acetyl-2-methylpyridine	1268.5	135.1	10.5 ± 2.8 ^a^	9.2 ± 0.5 ^a^
98	3,5-Dihydroxy-2-methyl-4H-pyran-4-one	1348.8	142	609 ± 216 ^a^	2767 ± 392 ^b^
99	1-(2-Hydroxy-5-methylphenyl)-ethanone	1471.2	150.1	75.5 ± 24.8 ^a^	108 ± 3.4 ^b^
100	1,2-Dihydro-3H-1,2,4-triazol-3-one	1485.7	85.1	27.1 ± 4.8 ^a^	106 ± 93.8 ^b^
101	2-Imidazolidinone	1502.7	86.1	42 ± 12.7 ^a^	184 ± 40.2 ^b^
102	4,4,6-Trimethyltetrahydro-1,3-oxazin-2-one	1518.5	128	51.9 ± 10.1 ^a^	167 ± 4.3 ^b^
103	(S)-4-Ethyl-2-oxazolidone	1531.6	85.1	41.8 ± 12.7 ^a^	160 ± 77.3 ^b^
104	1,3-Dioxol-2-one	1534.3	86.1	32.3 ± 8.5 ^a^	36.4 ± 16.3 ^a^
105	2,4,6-Tris(1,1-dimethylethyl)-4-methylcyclohexa-2,5-dien-1-one	1594.7	205.1	1.5 ± 1.7 ^a^	1.1 ± 0.3 ^a^
*Nitrogen compounds*					
106	1H-Imidazole-4-methanol	1025.7	98.1	4.5 ± 6.4 ^a^	46.7 ± 43.3 ^b^
107	N-Butyl-tert-butylamine	1149.5	114	16.6 ± 2.7 ^a^	59.6 ± 5.8 ^b^
108	3-Formyl-4,5-dimethyl-pyrrole	1162.2	123.1	n.d.	n.d.
109	5-Amino-2-methyl-2H-tetrazole	1273.5	71.1	11.9 ± 2.4 ^a^	47.4 ± 3.2 ^b^
110	2-Pyrrolidinone	1316.3	85.1	12.2 ± 1.9 ^a^	20.1 ± 18.4 ^a^
111	1-methyl-1H-pyrrole-2-carboxaldehyde	1343.2	109.1	20.2 ± 4.6 ^a^	94.8 ± 3.7 ^b^
112	1-Azabicyclo [3.1.0] hexane	1357.4	83.1	32.8 ± 31.2 ^a^	5.4 ± 4.3 ^b^
113	Succinimide	1417.2	99.1	7.7 ± 2.6 ^a^	23.9 ± 2.3 ^b^
114	Caprolactam	1491.7	113.1	3.2 ± 3.2 ^a^	4.3 ± 0.4 ^a^
115	m-Aminophenylacetylene	1532.4	117	0.9 ± 1.3 ^a^	10.6 ± 4.7 ^b^
116	(Z)-13-Docosenamide	3033.2	59.1	460 ± 100 ^a^	150 ± 43.3 ^b^
*Sulphur compounds*					
117	Dimethyl sulfone	1175.8	79.1	5.2 ± 1.6 ^a^	14.7 ± 0.9 ^b^
118	2,3-Dihydro-thiophene	1502.8	86.1	41.8 ± 12.7 ^a^	182 ± 39.3 ^b^
*Pyrans*					
119	2,3-Dehydro-1,8-cineole	1028.6	109.1	8.2 ± 2.6 ^a^	11.9 ± 0.7 ^a^
*Terpenes*					
120	3,7,11-Trimethyl-2,6,10-dodecatrien-1-ol	1853.7	69.1	12.2 ± 9.0 ^a^	4.8 ± 1.6 ^a^
121	Squalene	2854.7	69.1	33.3 ± 9.9 ^a^	24.3 ± 15.4 ^a^

n.d.: not detected; RI: retention index; *m*/*z*: mass-to-charge ratio.

**Table 3:** Odor threshold in water, relative odor activity value (OAV), and odor description of selected volatile compounds identified in peel and pulp of tamarillo.

No	Compounds	Odor Threshold in Water (ppb) ^a^	Relative Odor Activity Value (OAV)		Odor Description ^b^
			Peel	Pulp	
*Alcohols*					
1	2,3-Butanediol	30	16.39	56.41	Fruity, creamy
2	Alpha-terpineol	330–350	<1	<1	Floral, citrus, minty
3	Eugenol	6–30	9.94	1.78	Sweet
4	2,6-Dimethoxy-4-(2-propenyl)-phenol	1850	<1	<1	Sweet, spicy
*Aldehydes*					
5	3-Furaldehyde	3000	<1	1.44	Almond
6	Methional	0.2	77.12	588.59	Tomato, potato skin
7	Octanal	0.7	<1	52.34	Fatty, lemon, green
8	Nonanal	1	19.17	12.00	Fatty, citrus, green
9	(E)-2-Decenal	0.3–0.4	7.60	4.15	Fatty, green, mushroom
*Esters*					
10	Hexanoic acid, ethyl ester	1	5.55	11.80	Sweet, fruity, apple peel
11	Propanoic acid, 2-methyl-, ethyl ester	10	3.32	2.99	Sweet, fruity
12	Hexadecanoic acid, ethyl ester	2000	<1	<1	Fruity, creamy, waxy
*Fatty acids*					
13	Propanoic acid	20,000	<1	<1	Dairy, soy
14	Butanoic acid	240	<1	<1	Fruity, dairy, cheesy
15	Hexanoic acid	3000	<1	<1	Fatty, cheesy
16	Heptanoic acid	3000	<1	<1	Fruity, cheesy, pineapple
17	Octanoic acid	3000	<1	<1	Fatty, cheesy
18	Nonanoic acid	3000	<1	<1	Fatty, green
19	n-Decanoic acid	10,000	<1	<1	Fatty, citrus
20	Dodecanoic acid	10,000	<1	<1	Fatty, coconut, bay oil
21	Tetradecanoic acid	10,000	<1	<1	Fatty, pineapple citrus peel
22	Myristoleic acid	10,000	<1	<1	-
23	Z-11-Tetradecenoic acid	10,000	<1	<1	-
24	n-Hexadecanoic acid	10,000	<1	<1	Creamy
25	Octadecanoic acid	20,000	<1	<1	Fatty
*Furans*					
26	Furaneol	60	2.74	6.86	Fruity, caramel, burnt pineapple
27	5-Hydroxymethylfurfural	3000–230,000	<1	1.93	Caramel, buttery
*Hydrocarbons*					
28	1,1,5-Trimethyl-1,2-dihydronaphthalene	2	2.16	1.43	Licoricey, delicious wine
*Ketones*					
29	4-Hydroxy-4-methyl-2-pentanone	280	3.26	2.20	Minty
30	2-Hydroxy-3-methyl-2-cyclopenten-1-one	1000	<1	<1	Sweet, fruity
31	Acetophenone	65	<1	<1	Sweet, flower, almond
32	Maltol	35,000	<1	<1	Sweet, fruity caramel
*Nitrogen compounds*					
33	N-Butyl-tert-butylamine	50,000	<1	<1	-
34	Caprolactam	65	<1	<1	-
*Pyrans*					
35	2,3-Dehydro-1,8-cineole	12	<1	<1	Minty, lemon, sweet
*Terpens*					
36	3,7,11-Trimethyl-2,6,10-dodecatrien-1-ol (Farnesol)	20	<1	<1	Sweet, floral

^a^ Odor threshold values in water were adapted from Leffingwell & Associates’ website [15] and Wang, et al. [14]. ^b^ Odor descriptions of volatiles were adapted from Acree and Arn [16]. -: no data.

## Supplementary Materials

The following are available online at https://www.mdpi.com/article/10.3390/foods10092212/s1, Figure S1: Zone of inhibition testing with growth of (A) E. coli with ciprofloxacin and penicillin antibiotic discs; (B) E. coli with ciprofloxacin, amoxicillin, and penicillin antibiotic discs; and (C) Pseudomonas aeruginosa with ciprofloxacin, amoxicillin, and penicillin antibiotic discs, Figure S2: Inhibition zones caused by extracts of tamarillo peel on four bacteria (A: water extract, B: n-hexane extract, C: ethanol extract, D: methanol extract, E: control), Figure S3: Inhibition zones caused by extracts of tamarillo pulp on four bacteria (A: water extract, B: n-hexane extract, C: ethanol extract, D: methanol extract, E: control), Table S1: Comparison of volatile compounds between fresh and freeze-dried pulp of tamarillo using SPME–GC–MS (data presented as percentage of relative concentration), Table S2: Comparison of volatile compounds between fresh and freeze-dried peel of tamarillo using SPME–GC–MS (data presented as percentage of relative concentration).
