# Supplementary material for: Volatile Components and Preliminary Antibacterial Activity of Tamarillo (Solanum betaceum Cav.)
Source: Foods. 2021 Sep 17;10(9):2212. doi: 10.3390/foods10092212 (PMC8470738; doi:10.3390/foods10092212)
Supplement: Supplementary file 1 [file foods-10-02212-s001.zip › foods-1379790-supplementary.pdf]

Table S1. Comparison of volatile compounds between fresh and freeze-dried pulp of tamarillo using SPME-GC-MS (data presented as percentage of relative concentration).

No	Compounds	Retention index	Fresh (%)	Freeze-dried (%)	% (Freeze-dried – Fresh)/ Freeze-dried	
1	3-methyl-Butanal	747	0.017	0.086	80.7	
2	1-Methoxy-3-methyl-3-butene	761	0.898	0.137		- 84.7
3	Butanoic acid, methyl ester	788	10.146	8.470		- 16.5
4	1-methyl-1,4-Cyclohexadiene	791	1.145	0.260		- 77.3
5	Methyl isovalerate	835	0.241	0.154		- 36.1
6	3-methyl-3-Buten-1-ol	842	1.861	0.909		- 51.2
7	Butanoic acid, ethyl ester	860	0.018	0.548	96.7	
8	3-Butenoic acid, 3-methyl-, methyl ester	866	1.614	0.476		- 70.5
9	1-Pentanol	876	0.026	0.122	78.9	
10	Hexanal	881	1.443	0.949	34.2	
11	3-Hexenal	884	0.522	0.323		- 38.2
12	Prenol	888	0.417	0.390		- 6.5
13	Isopropyl butyrate	899	0.263	0.127		- 51.8
14	2-Ethyl-tetrahydropyran	900	0.165	0.145		- 11.9
15	2-methyl-2-Butenal	902	0.557	0.729	23.6	
16	2-Butenoic acid, 3-methyl-, methyl ester	914	7.818	2.624		66.4
17	(S)-(+)-1,2-Propanediol (Tentative)	936	n.d	1.194		
18	4-methyl-1-(1-methylethyl)-Bicyclo[3.1.0]hex-2-ene	947	0.110	0.109		- 1.7
19	1-Butanol, 2-methyl-, acetate	941	0.037	0.032		- 13.9
20	Alpha-Pinene	947	0.067	0.145	54.0	
21	2,2-dimethyl- 3-Octene (Tentative)	951	0.091	0.093	2.0	
22	3-Methyl-3-buten-1-ol, acetate	958	15.543	6.633		- 57.3
23	2-Hexenal	962	3.494	3.187		- 8.8
24	[R-(R*,R*)]-2,3-Butanediol	968	n.d	16.426		
25	(Z)- 3-Hexen-1-ol	970	1.802	1.610		- 10.6
26	[S-(R*,R*)]-2,3-Butanediol	976	n.d	0.668		
27	Propyl-Cyclopropane	979	3.926	0.675		- 82.8
28	Hexanoic acid, methyl ester	991	30.200	36.926	18.2	
29	2-Buten-1-ol, 3-methyl-, acetate	996	1.860	0.745		- 60.0
30	2,3-Dehydro-1,8-cineole	1034	0.596	2.225	73.2	
31	Ether, 2-ethylhexyl tert-butyl	1054	0.014	0.552	97.5	
32	D-Limonene	1055	0.025	0.101	75.6	
33	Hexanoic acid, ethyl ester	991	0.025	0.505	95.0	
34	3-Methyl-3-butenoic acid	1070	0.017	0.647	97.4	
35	o-Cymene	1073	0.171	0.336	49.2	
36	Eucalyptol	1075	1.292	0.496		- 61.6
37	(Z)- 3-Hexen-1-ol, acetate	1080	0.150	0.064		- 57.4
38	2-methyl-3-(1-methylethenyl)-, (1a,2a,3a)- Cyclohexanol (Tentative)	1083	0.046	0.138	66.8	

39	Pentanoic acid, 2-hydroxy-3-methyl-, methyl ester	1097	0.030	0.163	81.5	
40	.+/-.-Tetrahydro-3-furanmethanol	1115	n.d	0.714		
41	Butanoic acid, 4-pentenyl ester	1135	10.474	2.641		- 74.8
42	Butanoic acid, 3-methylbut-2-enyl ester	1169	0.239	0.062		- 74.2
43	1-methyl-2-octyl-Cyclopropane,	1177	0.026	0.110	76.3	
44	Octanoic acid, 3-hydroxy-, methyl ester	1181	0.008	0.104	92.3	
45	Butanedioic acid, methyl-, dimethyl ester	1186	0.016	0.245	93.4	
46	Octanoic acid, methyl ester	1190	0.284	0.204		- 28.2
47	Nonanal	1192	0.051	0.265	80.9	
48	Linalool	1194	0.024	0.010		- 60.2
49	Ethylene glycol di-n-butyrate (Tentative)	1198	0.125	0.237	47.1	
50	Benzoic acid, hydrazide	1200	0.417	0.499	16.5	
51	Hexanoic acid, 4-oxo-, methyl ester	1230	0.124	1.863	93.3	
52	(Z)- Butanoic acid, 3-hexenyl ester	1255	0.007	0.042	82.8	
53	5-ethyldihydro-2(3H)-Furanone (Tentative)	1262	0.052	0.485	89.3	
54	2-Acetyl-5-methylfuran (Tentative)	1264	0.014	0.172	91.7	
55	[R-(R*,R*)]-1,2-diphenyl-, 1,2-Ethanediol (Tentative)	1265	0.031	0.070	56.1	
56	Terpinen-4-ol	1267	0.063	0.017		- 73.8
57	4-methyl-1-Hexanol	1276	0.251	0.086		- 65.7
58	(E,E)-2,6-Nonadienal	1276	0.251	0.086		- 65.7
59	Butyric acid, 2-hydroxy-3-methyl-, methyl ester	1277	0.005	0.219	97.9	
60	p-Mentha-1,5-dien-8-ol	1285	0.062	0.269	76.9	
61	L-.alpha.-Terpineol	1298	0.399	0.201		- 49.7
62	Methyl salicylate	1305	0.071	0.012		- 82.7
63	p-Mentha-1,5-dien-8-ol (Tentative)	1322	0.110	0.595	81.5	
64	1,3,3-trimethyl- 2-Oxabicyclo[2.2.2]octan-6-ol,	1327	0.001	0.098	98.5	
65	Hexanoic acid, 4-pentenyl ester	1328	0.068	0.025		- 63.6
66	1,3,3-trimethyl-2-Oxabicyclo[2.2.2]octan-6-one	1348	0.026	0.042	37.8	
67	2,6,6-trimethyl- Bicyclo(3.1.1)heptane-2,3-diol	1348	n.d	0.086		
68	Butanoic acid, 2,3-dihydroxypropyl ester	1367	0.030	0.030	2.2	
69	(Z)- 2-methoxy-4-(1-propenyl)-Phenol	1383	0.049	0.012		- 75.9
70	Methyleugenol	1532	0.007	0.0002		- 97.4
71	2,6-Dimethyl-2-trans-6-octadiene (Tentative)	1551	0.014	0.208	93.4	
72	l-Alanine, N-(2,3,4-trifluorobenzoyl)-, methyl ester (Tentative)	1587	0.002	0.011	84.5	
73	Aristol-1(10)-en-9-yl isovalerate	1726	0.029	0.122	76.5	

Table S2. Comparison of volatile compounds between fresh and freeze-dried peel of tamarillo using SPME-GC-MS (data presented as percentage of relative concentration).

No.	Compounds	Retention index	Fresh (%)	Freeze-dried (%)	% (Freeze-dried – Fresh)/Freeze-dried	
1	3-methyl-Butanal,	747	0.018	0.278	93.6	
2	2-methyl-Butanal,	751	0.136	0.776	82.5	
3	1-Penten-3-one	777	n.d	0.154		
4	Pentanal	781	0.022	0.479	95.3	
5	Butanoic acid, methyl ester	787	9.991	2.414		- 75.8
6	1-methyl-1,4-Cyclohexadiene,	797	3.797	2.167		- 42.9
7	3-methyl-3-Buten-1-ol,	840	3.330	1.409		- 57.7
8	3-Penten-1-ol	840	3.035	1.403		- 53.8
9	Hexane, 2,2,5-trimethyl- (Tentative)	848	1.234	0.108		- 91.3
10	Butanoic acid, ethyl ester	862	2.201	0.093		- 95.8
11	Hexanal	881	0.195	0.990	80.3	
12	Prenol	888	0.588	0.449	23.6	
13	Tetrahydro-2H-Pyran-2-methanol, (Tentative)	900	0.052	0.317	83.7	
14	Isopropyl butyrate	900	0.036	0.033		- 7.0
15	3-methyl-2-Butenal,	903	0.543	0.125		- 77.0
16	2-Butenoic acid, 3-methyl-, methyl ester	915	1.670	0.707		- 57.7
17	Propylene Glycol (Tentative)	936	n.d	0.788		
18	4-methyl-1-(1-methylethyl)-Bicyclo[3.1.0]hex-2-ene	943	3.431	0.371		- 89.2
19	7-methylene-Bicyclo[4.1.0]heptane	948	1.295	0.930		- 28.2
20	5-Chloro-5-methylnonane (Tentative)	950	0.008	0.088	90.6	
21	3-Methyl-3-buten-1-ol, acetate	957	0.111	2.627	95.8	
22	(E)-2-Hexenal	961	0.084	2.763	97.0	
23	[S-(R*,R*)]-2,3-Butanediol	967	n.d	6.728		
24	(E)-3-Hexen-1-ol	972	36.246	29.439		- 18.8
25	1-Hexanol	978	10.182	4.414		- 56.6
26	Hexanoic acid, methyl ester	989	6.105	14.936	59.1	
27	2-Buten-1-ol, 3-methyl-, acetate	996	n.d	0.465		
28	ß-Pinene	1000	0.748	0.464		- 38.0
29	.beta.-Phellandrene	1002	2.805	0.450		- 83.9

30	2,2-dimethyl-1-Butanol	1026	0.062	0.194	68.2	
31	1-(6,7,7-trimethyl-2,3-Tricyclo[2.2.1.0(2,6)]heptane, 1,7,7-trimethyl-	1031	0.043	0.152	71.4	
32	2-Buten-1-one, [1R-[1.alpha.(E),4.beta.]]-dioxabicyclo[2.2.2]oct-5-en-1-yl)-	1032	n.d	0.099		
33	2,3-Dehydro-1,8-cineole	1033	0.710	1.129	37.0	
34	2,3-Dehydro-1,8-cineole (isomer?)	1040	0.008	0.032	75.0	
35	8-methyl-1,8-Nonanediol (Tentative)	1041	0.086	0.210	58.9	
36	pentyl-Cyclopentane	1044	n.d	0.045		
37	1-methyl-4-(1-methylethylidene)-Cyclohexene	1046	0.288	0.032		- 89.0
38	1,2,4-trimethyl-Benzene	1048	0.226	0.334	32.4	
39	2,3-Dimethyldecane (Tentative)	1050	n.d	0.092	99.8	
40	tert-Butyl glycidyl ether	1051	0.002	2.196	99.9	
41	Ether, 2-ethylhexyl tert-butyl	1055	0.013	5.491	99.8	
42	D-Limonene	1055	0.662	0.839	21.1	
43	Hexanoic acid, ethyl ester	1055	0.558	0.217		- 61.1
44	p-Cymene	1062	1.851	0.357		- 80.7
45	Eucalyptol	1075	0.353	1.346	73.8	
46	(Z)-3-Hexen-1-ol, acetate	1080	0.699	0.477		- 31.7
47	ß-Ocimene	1083	0.101	0.077		- 23.2
48	?-Terpinene	1088	0.481	0.063		- 87.0
49	Octanal	1089	0.004	0.287	98.5	
50	(E)- 9-methyl-3-Undecene (Tentative)	1126	n.d	0.878		
51	Butanoic acid, 4-pentenyl ester	1135	1.249	3.191	60.9	
52	Ethyl 2-(5-methyl-5-vinyltetrahydrofuran-2-yl)propan-2-yl carbonate	1156	n.d	0.108		
53	5-methyl-6-methylene-Decane,	1177	0.029	0.451	93.5	
54	Octanoic acid, methyl ester	1190	0.460	0.263		- 42.7
55	Nonanal	1192	0.015	0.316	95.3	
56	Linalool	1194	0.072	0.036		- 49.7
57	(E)- 3-methyl-2-Undecene	1195	0.009	0.616	98.5	
58	2-methyl-Undecane(Tentative)	1216	n.d	0.142		
59	Carbonic acid, bis(2-ethylhexyl) ester	1220	n.d	0.173		
60	5-methyl-5-propyl-Nonane (Tentative)	1227	n.d	0.150		
61	Hexanoic acid, 4-oxo-, methyl ester	1230	0.010	0.487	97.9	
62	2,6-dimethyl-Cyclohexanol	1231	0.010	0.091	89.0	
63	Methyl 1-acetylpyrrolidine-2-carboxylate (Tentative)	1232	0.003	0.300	98.9	
64	1-chloro-4-methoxy-Benzene	1235	2.159	0.021		- 99.0
65	Dodecyl pentyl ether (Tentative)	1249	0.202	0.561	63.9	
66	(Z)-Butanoic acid, 3-hexenyl ester,	1255	1.144	0.136		- 88.1
67	2,3,4-trimethyl-Thiophene, (Tentative)	1265	0.002	0.222	98.9	
68	alpha-Terpineol	1298	0.368	0.336		- 8.6
69	2,6,11,15-tetramethyl-Hexadecane,	1307	n.d	0.030		
70	3,7-dimethyl-Nonane(Tentative)	1316	n.d	0.076		

71	p-Mentha-1(7),2-dien-8-ol	1322	0.057	0.075	23.8
72	Methacrylic acid, hexadecyl ester (Tentative)	1335	n.d	0.066	
73	1,3,3-trimethyl-2-Oxabicyclo[2.2.2]octan-6-one	1348	0.066	0.160	58.8
74	1-(2-hydroxy-5-methoxyphenyl)-Ethanone(Tentative)	1361	n.d	0.029	
75	2,6,6-trimethyl-Bicyclo(3.1.1)heptane-2,3-diol(Tentative)	1367	0.018	0.112	83.8
76	2,6,10,15-tetramethyl-Heptadecane(Tentative)	1375	n.d	0.029	
77	2,6,10-trimethyl-Tetradecane(Tentative)	1403	n.d	0.051	
78	2-octyl-1-Decanol(Tentative)	1421	n.d	0.018	
79	6-propyl-Tridecane(Tentative)	1433	n.d	0.010	
80	2,6,10-trimethyl-Tetradecane(Tentative 2)	1449	n.d	0.030	
81	Nonyl-Cyclopentane (Tentative)	1455	0.004	0.063	93.8
82	2,6,10-trimethyl-Tetradecane	1458	0.011	0.013	12.3
83	(Z)-Methyl heptadec-9-enoate (Tentative)	1499	n.d	0.021	
84	3-Allyl-6-methoxyphenol	1506	0.013	0.122	89.2
85	[2a(R*),3a]-(.+-.)-Cyclohexanone, 2,2-dimethyl-5-(3-methyloxiranyl)-	1551	0.054	0.385	86.0
86	(E)- 6,10-dimethyl-5,9-Undecadien-2-one	1562	0.006	0.011	47.4
87	4-(2,6,6-trimethyl-1-cyclohexen-1-yl)-3-Buten-2-one,	1620	0.025	0.102	75.8
88	4-(2,2,6-trimethyl-7-oxabicyclo[4.1.0]hept-1-yl)-3-Buten-2-one	1651	0.003	0.088	96.8

(A) (B) (C)

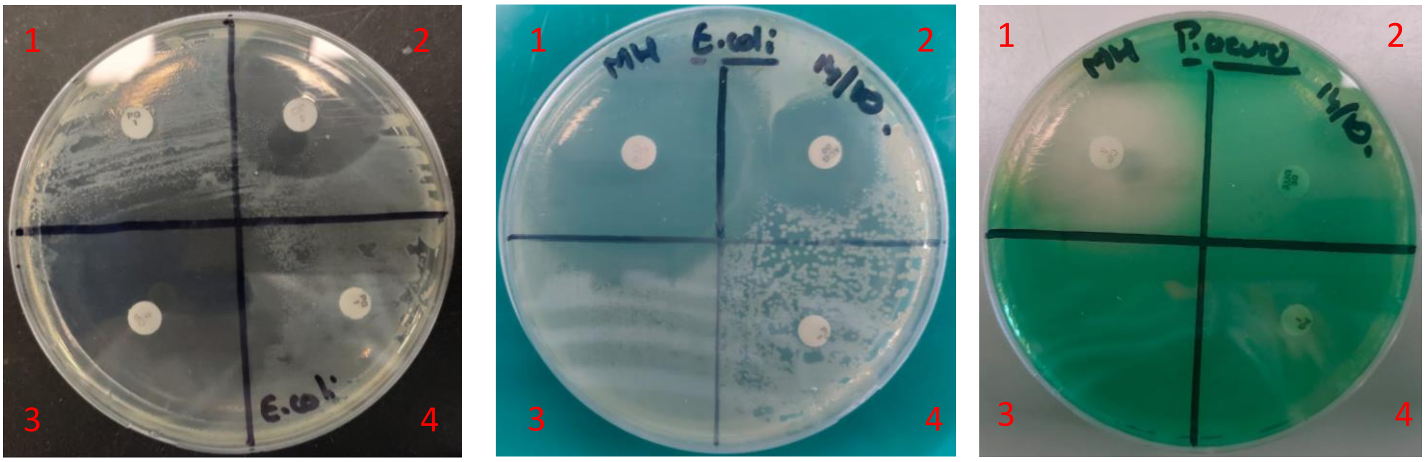

Figure S1. Zone of inhibition testing with growth of (A) *E. coli* with ciprofloxacin (#2, 3) and penicillin (#1, 4) antibiotic discs; (B) *E. coli* with ciprofloxacin (#1), amoxicillin (#2), and penicillin (#3) antibiotic discs. #4 did not have any antibiotic disc (control); and (C) *Pseudomonas aeruginosa* with ciprofloxacin (#1), amoxicillin (#2), and penicillin (#4) antibiotic discs. #3 did not have antibiotic disc (control)

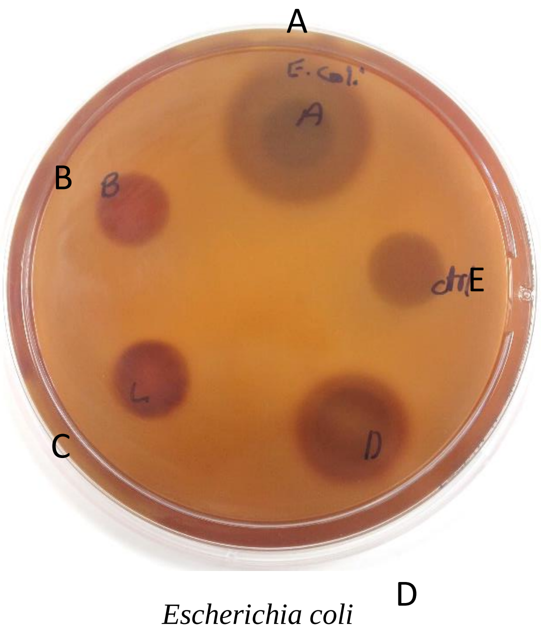

Escherichia coli

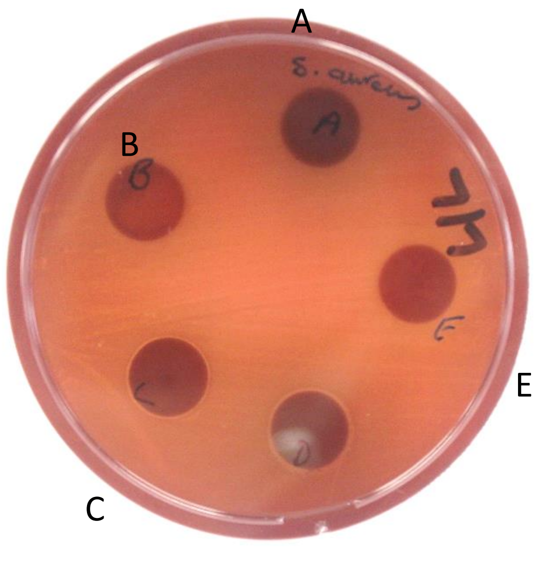

Staphylococcus aureus

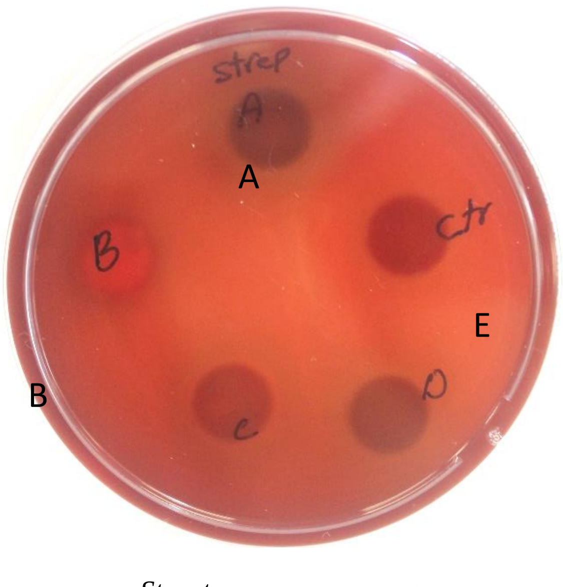

Streptococcus pyogenes

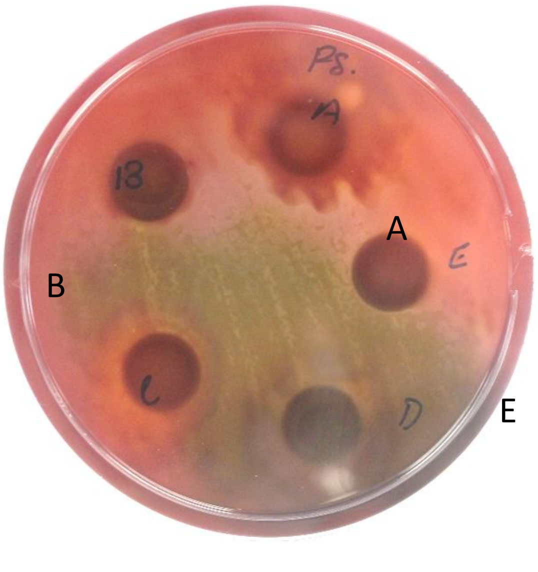

Pseudomonas aeruginosa

C

C D D

Figure S2. Inhibition zones caused by extracts of tamarillo peel on four bacteria (A: water extract, B: n-hexane extract, C: Ethanol extract, D: Methanol extract, E: Control)

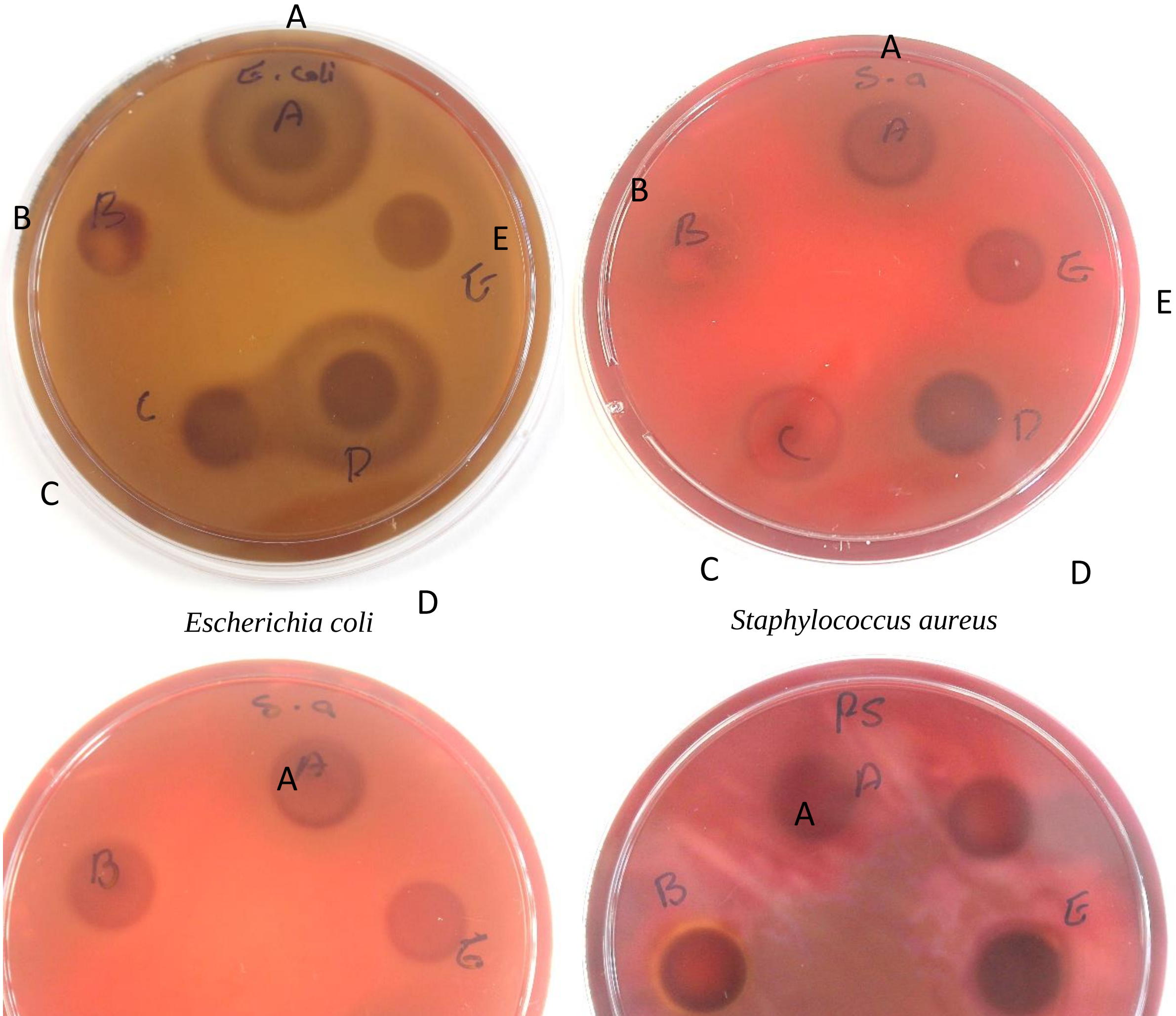

Escherichia coli

Staphylococcus aureus

E

B E B

D

c

D c

Figure S3. Inhibition zones caused by extracts of tamarillo pulp on four bacteria (A: water extract, B: n-hexane extract, C: Ethanol extract, D: Methanol extract, E: Control).
